# Delayed large pruritic eruption to the BNT162b2 mRNA vaccine

**DOI:** 10.1016/j.idcr.2021.e01297

**Published:** 2021-09-25

**Authors:** Takeshi Terashima

**Affiliations:** Department of Respiratory Medicine, Tokyo Dental College Ichikawa General Hospital, 5-11-13 Sugano, Ichikawa, Chiba 272-0824, Japan

**Keywords:** Delayed cutaneous reaction, mRNA vaccine, BNT162b2, Elderly

The incidence of delayed cutaneous reactions to the SARS-CoV-2 mRNA-1273 vaccine has been reported at 0.8% following the initial dose. The mean age of those exhibiting these reactions is 43 years. The median onset is 8 days post-vaccination, with resolution typically at a median of 6 days post-onset [1]. Although the “COVID arm” has been documented, there are only few detailed reports of it resulting from the administration of the BNT162b2 vaccine [2]. We present a case of such a reaction to BNT162b2 in an elderly patient.

A 75-year-old woman with no prior allergy history exhibited a large, pruritic eruption (60 × 35 mm) 5 days following her first dose of the vaccine (Fig. 1). It was accompanied neither by pain nor induration and resolved spontaneously 6 days following onset. She was encouraged to complete the course, following which there was no recurrence. This report documents that delayed cutaneous reactions may follow BNT162b2 administration, even among elderly patients, but should not discourage vaccination.

**Fig. 1:**
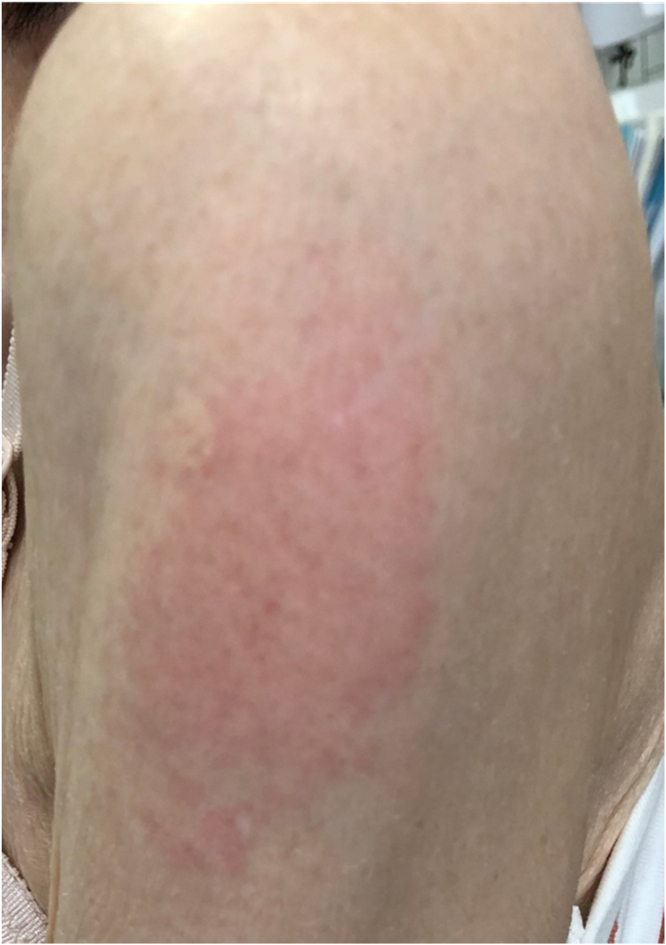
A large, pruritic eruption on the arm following the first dose of BNT162b2 vaccine.

## Ethical approval

This study was approved by the ethics committee of Tokyo Dental College.

## Consent

Written informed consent was obtained from the patient for publication of this case report and accompanying images. A copy of the written consent is available for review by the Editor-in-Chief of this journal on request.

## Funding

This research did not receive any specific grant from funding agencies in the public, commercial, or not-for-profit sectors.

## Author contribution

TT contributed to the collection of clinical data, data analysis, and manuscript writing. All authors have read and approved the final manuscript.
